# Insights into the computer-aided drug design and discovery based on anthraquinone scaffold for cancer treatment: A systematic review

**DOI:** 10.1371/journal.pone.0301396

**Published:** 2024-05-22

**Authors:** Hui Ming Chua, Said Moshawih, Nurolaini Kifli, Hui Poh Goh, Long Chiau Ming

**Affiliations:** 1 PAP Rashidah Sa’adatul Bolkiah Institute of Health Sciences, Universiti Brunei Darussalam, Gadong, Brunei Darussalam; 2 School of Medical and Life Sciences, Sunway University, Bandar Sunway, Malaysia; School of Pharmacy - Bandung Institute of Technology, INDONESIA

## Abstract

**Background:**

In the search for better anticancer drugs, computer-aided drug design (CADD) techniques play an indispensable role in facilitating the lengthy and costly drug discovery process especially when natural products are involved. Anthraquinone is one of the most widely-recognized natural products with anticancer properties. This review aimed to systematically assess and synthesize evidence on the utilization of CADD techniques centered on the anthraquinone scaffold for cancer treatment.

**Methods:**

The conduct and reporting of this review were done in accordance to the Preferred Reporting Items for Systematic Reviews and Meta-analysis (PRISMA) 2020 guideline. The protocol was registered in the “International prospective register of systematic reviews” database (PROSPERO: CRD42023432904) and also published recently. The search strategy was designed based on the combination of concept 1 “CADD or virtual screening”, concept 2 “anthraquinone” and concept 3 “cancer”. The search was executed in PubMed, Scopus, Web of Science and MedRxiv on 30 June 2023.

**Results:**

Databases searching retrieved a total of 317 records. After deduplication and applying the eligibility criteria, the final review ended up with 32 articles in which 3 articles were found by citation searching. The CADD methods used in the studies were either structure-based alone (69%) or combined with ligand-based methods via parallel (9%) or sequential (22%) approaches. Molecular docking was performed in all studies, with Glide and AutoDock being the most popular commercial and public software used respectively. Protein data bank was used in most studies to retrieve the crystal structure of the targets of interest while the main ligand databases were PubChem and Zinc. The utilization of in-silico techniques has enabled a deeper dive into the structural, biological and pharmacological properties of anthraquinone derivatives, revealing their remarkable anticancer properties in an all-rounded fashion.

**Conclusion:**

By harnessing the power of computational tools and leveraging the natural diversity of anthraquinone compounds, researchers can expedite the development of better drugs to address the unmet medical needs in cancer treatment by improving the treatment outcome for cancer patients.

## Introduction

The public health burden of cancer is rising rapidly. The American Cancer Society estimates that in year 2023 alone, nearly 2 million new cancer cases and half a million of cancer deaths will occur in the United States [1]. At the global level, 28.4 million of cancer cases is projected in year 2040 by the International Agency for Research on Cancer (IARC), translated to a 47% increment in 20 years of time [2]. Despite the remarkable achievements in oncology research for the past few decades that are extending the lives of many patients, there are still unmet medical needs due to resistance and relapse after a certain time of treatment [3]. On the other hand, almost all types of cancer treatment modalities cause different degrees of side effects, jeopardizing the patients’ quality of life. For instance, conventional cancer therapies frequently result in organ toxicity leading to long term complications, and even the more advanced remedies such as immunotherapy may cause serious or deadly allergic reactions [4]. Therefore, the search for better anticancer drug with a good balance in between efficacy and safety continues to attract the attention of researchers.

It is an established fact that the journey to discover a novel drug is long, costly and fraught with challenges. A recent systematic review revealed that the research and development (R&D) cost of a new molecular entity could reach USD4.54 billion in estimation, with anticancer drug being the most expensive therapeutic to make [5]. Worst still, huge investment cost does not guarantee success in bringing one new medication from bench to bed due to the high attrition rate especially at the late stage (non-clinical and clinical trials) of the drug development process [6, 7]. It is crucial to improve the productivity of R&D and computer-aided drug design (CADD) comes right into the scene to facilitate this endeavour. CADD makes use of different software, mathematical models and algorithms to rationalize the drug design and speed up the drug discovery process especially at the early phases which include target identification, hit identification, hit-to-lead and lead optimization [8]. With the aid of in-silico tools, the number of chemical candidates to be tested in-vitro or in-vivo are greatly reduced, the success rate of clinical trials is also increased, leading to the optimization of resources and enhanced cost-effectiveness throughout the trajectory of drug discovery and development [9]. The advantages of CADD are also evident in discovering novel drug to tackle allosteric cancer targets or management of tumours that formed through complicated pathways [10].

In general, CADD can be divided into either structure-based or ligand-based approaches. As the name implies, structure-based methods rely on the availability of three-dimensional (3D) structure of the macromolecular target. Whereas ligand-based methods require the information of at least one chemical compound of interest or a set of known actives to begin with [9]. When the drug design and discovery process involved screening a set of compounds or chemical databases to pinpoint promising hits by utilizing computer software and algorithm, the term is coined as ‘virtual screening”. Likewise, structure-based virtual screening (SBVS) and ligand-based virtual screening (LBVS) are the common categories to differentiate between the involvement of either target molecules or known active compounds as the starting point in the screening process. Both strategies can be used independently or in a hybrid manner for virtual screening [11].

The most established structure-based CADD tool is molecular docking which predicts the interactions and degree of complementary between the ligands and the target’s binding site [12]. Through docking-based virtual screening, potential hits can be shortlisted from the vast chemical space based on the scoring ranked by the docking software [7]. The more negative the scoring value, the tighter the binding of the ligand-target is deduced [13]. Structure-based pharmacophore modelling is another technique that utilizes the information gathered from the binding sites of target structures to generate a molecular framework that outlines the essential features required for binding, followed by virtual screening to map for potential binders from the chemical database [14].

Pharmacophore is defined as the ‘ensemble of steric and electronic features that is required to ensure optimal interactions with a target of interest or to exert its biological response (either by activating or inhibiting it). Pharmacophoric descriptors are used to define a pharmacophore, including hydrogen-bonding (acceptors or donors), hydrophobic groups, electrostatic interaction sites (positively or negatively ionizable groups), ring centres (aromatic groups) and virtual points (metal coordinating areas) [15]. When the structural information of the macromolecule target is unavailable, the physicochemical properties of a set of known actives are used to perform pharmacophore modelling and mapping instead [14]. This is one example of the ligand-based approach, in which molecular descriptors known to be essential for biological activities are gathered to retrieve other potential drug candidates based on the similarity principle that indicates similar molecules normally carry similar activities [16]. Other examples of ligand-based CADD methods include similarity search, scaffold hopping and quantitative-structure-activity relationship (QSAR).

After docking-based virtual screening, molecular dynamic simulation can be used to visualise the movement and interaction of ligand-target complex over time by simulating dynamical changes in the system. By analysing the snapshots taken throughout the simulation time, flexibility and stability of the ligand-target complex can be predicted, location of water molecules or change in entropy of special structures can also be observed. These hidden states of the system can by no means be tested by any wet-lab technique [7, 17]. The molecular mechanics energies combined with the Poisson-Boltzman (MM-PBSA) or generalized Born and surface area continuum solvation (MM-GBSA) are other commonly used tool for post-docking analysis to estimate the free binding energy of the ligand-target complex [18]. Both molecular dynamic simulation and MM-GBSA/PBSA have been shown to successfully improve the results of virtual screening and are particularly useful in the lead optimization stage [18, 19].

CADD tools are also useful to predict the pharmacokinetics properties and toxicology profile of potential drug candidates, in which various types of in-silico ADMET (adsorption-distribution-metabolism-excretion and toxicity) filters are available to remove compounds that carry undesired properties either before or after virtual screening. These web-based filters are being used extensively to finetune the virtual chemical database or combinatorial libraries, as well as during the lead optimization stage to enhance the pharmacological properties of the lead compounds and subsequently increase the success rate at the downstream stages of the drug discovery process [20].

Apart from that, making use of computational tools to build a combinatorial library for virtual screening is also getting popular in drug discovery. The combinatorial library refers to a set of new compounds prepared by a single stepwise enumeration of existing ligands using different types of substitution [21]. With today’s advancement in computational power, only a few seconds are needed to construct a virtual combinatorial library with millions of compounds [22]. Combinatorial library has been used in natural product research to create databases of natural product analogues with drug-like properties [23]. This strategy can uncover the potential of natural products with privileged scaffold for new drug design and discovery, for instance, the anthraquinone- and chalcone- derivatives that showed a wide spectrum of biological effects on many different macromolecular targets responsible for human diseases including cancer were used to construct virtual library as the starting point of new drug research [24].

Natural products and their derivatives have a long history in the pharmaceutical world owing to their rich bioactive constituents with remarkable therapeutic potential and contributed to the discovery of many new chemical entities especially in the early days [25]. Medicinal plants that worked in both minor ailments and severe illnesses including cancer are cheaper and cause lesser side effects as compared to pure chemical drugs [26]. Combining natural products with chemotherapeutics has been shown to provide a synergistic effect and overcome many of the chemo-resistance hurdles in cancer treatment [27].

Anthraquinone is one of the most widely-recognized natural products with great medicinal value especially in the oncology setting as evidenced in many of the published reviews [28–33]. There have been many pieces of research characterized and studied anti-cancer properties of naturally occurring anthraquinone derivatives in different cancer types, for example, emodin in leukaemia [34], colorectal cancer [35] and breast cancer [36]; aloe-emodin in oral cancer [37], lung cancer [38] and neuroectodermal cancer [39]; chrysophanol in liver cancer [40] and many others. Well-established cytotoxic drugs like doxorubicin and mitoxantrone also contain anthraquinone moiety [41]. This basic scaffold of anthraquinone is made up of two aromatic rings that are connected by two carbonyl groups (mostly at positions 9 and 10) to form a planar structure (S1 Fig). This unique scaffold has attracted intense interest in the research endeavours towards designing anthraquinone-derived medicines for many different medical conditions including malignancy. Typical molecular targets of anthraquinone derivatives in cancer therapeutics include enzymes that are involved in cellular signalling pathways such as topoisomerase and kinases, DNA intercalation, inflammatory processes and redox reactions [32]. Having a planar aromatic structure enables anthraquinone derivatives to intercalate with DNA or interact with DNA topoisomerases, leading to an interruption of the transcription and replication process. Likewise, interaction with kinases can also disrupt cellular differentiation and repair function, resulting in apoptosis of cancer cells [42]. On the other hand, the quinone moiety in anthraquinone exhibits antioxidant properties by acting as reactive oxygen species (ROS) regulators [43]. Although there have been a considerable amount of reviews done on the topic of the anticancer potential of anthraquinone, and there was also a recent publication discussed the past, present and future role of computer-aided drug discovery in cancer research [44], however none of these reviews linked together the role of virtual screening in facilitating the drug design and discovery in cancer treatment based on anthraquinone scaffold. More importantly, none of them are systematic reviews. To the best of our knowledge, this is the first systematic review synthesizing the evidence of computer-aided drug design and discovery based on an anthraquinone scaffold for the treatment of cancer.

The primary objective of this review was to systematically assess and synthesize evidence on the utilization of computer-aided drug design (CADD) techniques centered on the anthraquinone scaffold for cancer treatment. We highlight recent trends, popular computational methodologies, preferred software tools, and databases in discovering anticancer drugs that involved anthraquinone scaffolds. Additionally, the therapeutic potential of these anthraquinone derivatives across various cancer types is summarized together with the identified target proteins that tackled multiple malignancy pathways. Our findings intend to provide a robust foundation for future research, offering evidence-based insights for successful drug design leveraging the CADD techniques and anthraquinone scaffold.

## Materials and methods

### Study protocol

This systematic review was conducted and reported based on the Preferred Reporting Items for Systematic Reviews and Meta-analysis (PRISMA) 2020 guideline [45, 46]. The protocol was published [47] and registered in the “International prospective register of systematic reviews” database (PROSPERO: CRD42023432904).

### Review question

The review was conducted to answer the main research questions as follows:

“What are the trends and types of computer-aided drug design and discovery tools used in virtual screening based on anthraquinone scaffold for cancer treatment”?

“What are the therapeutic potential and target protein of anthraquinone and derivatives elucidated by CADD to treat cancer?

### Eligibility criteria

The review question and eligibility criteria were established according to the PECo strategy (P, problem; E, exposure; Co, context) for systematic review. Only original research studies that were published in English and utilized CADD tools as the primary method to discover or design anticancer drugs involving compounds with anthraquinone scaffold were included in the review. The details of the inclusion and exclusion criteria based on the PECo strategy are outlined in Table 1.

**Table 1:** Eligibility criteria based on the PECo strategy.

Element	Inclusion Criteria	Exclusion Criteria
Problem (P)	Studies with clear descriptions of the CADD tools used in virtual screening involving compounds with anthraquinone scaffold were included.	Studies without details or clear descriptions of the CADD or virtual screening tools and studies not involving compounds with anthraquinone scaffold were excluded.
Exposure (E)	Studies investigating therapeutic potential and target protein of compounds involving anthraquinone scaffold for cancer treatment were included.	Studies investigating diseases other than cancer and not involving anthraquinone derivatives were excluded.
Context (Co)	Only original research studies published in English and utilized CADD techniques or virtual screening tools for either target protein prediction/ validation, hit identification, hit-to-lead and lead optimization were included.	Studies exclusively in-vitro, in-vivo or other types of in-silico tools that did not serve the purpose of target protein prediction/validation, hit identification, hit-to-lead or lead optimization were excluded. Network pharmacology was excluded as it did not align with the scope of this review. Review article, book chapter, letters, grey literatures (conference paper abstracts, theses/ dissertation, report) and articles published in languages other than English were excluded.

### Information sources and search strategy

On 30 June 2023, the literature search was executed on four electronic databases which include PubMed, Scopus, Web of Science and MedRxiv. There was no restriction on the publication period, but only articles published in the English language were saved. The search strategy was designed based on the combination of three main concepts, namely CADD or virtual screening (Concept 1), anthraquinone (Concept 2) and cancer (Concept 3). The aim was to retrieve studies that used CADD tools such as molecular docking, molecular dynamic simulation or any other virtual screening method as the primary approach in the quest for anticancer drugs involving anthraquinone scaffold. The main search string was as follows: (“virtual screening” OR “computer aided drug design” OR “molecular docking” OR “molecular dynamics”) AND (“anthraquinone” OR “anthracenedione” OR “anthranoid” OR “anthradione” OR “dioxoanthracene” OR “anthracene-9,10-dione” OR “anthracene-9,10-quinone” OR “9,10-anthrachinon” OR “9,10-dihydro-9,10-dioxoanthracene”) AND (cancer OR tumour OR malignant OR neoplasm). The search was focused on the title, abstract and keywords of the articles and adjustments were made in each database based on their different characteristics. The S1 Table tabulates the search strategy executed in the PubMed database.

### Study selection

**Fig 1:**
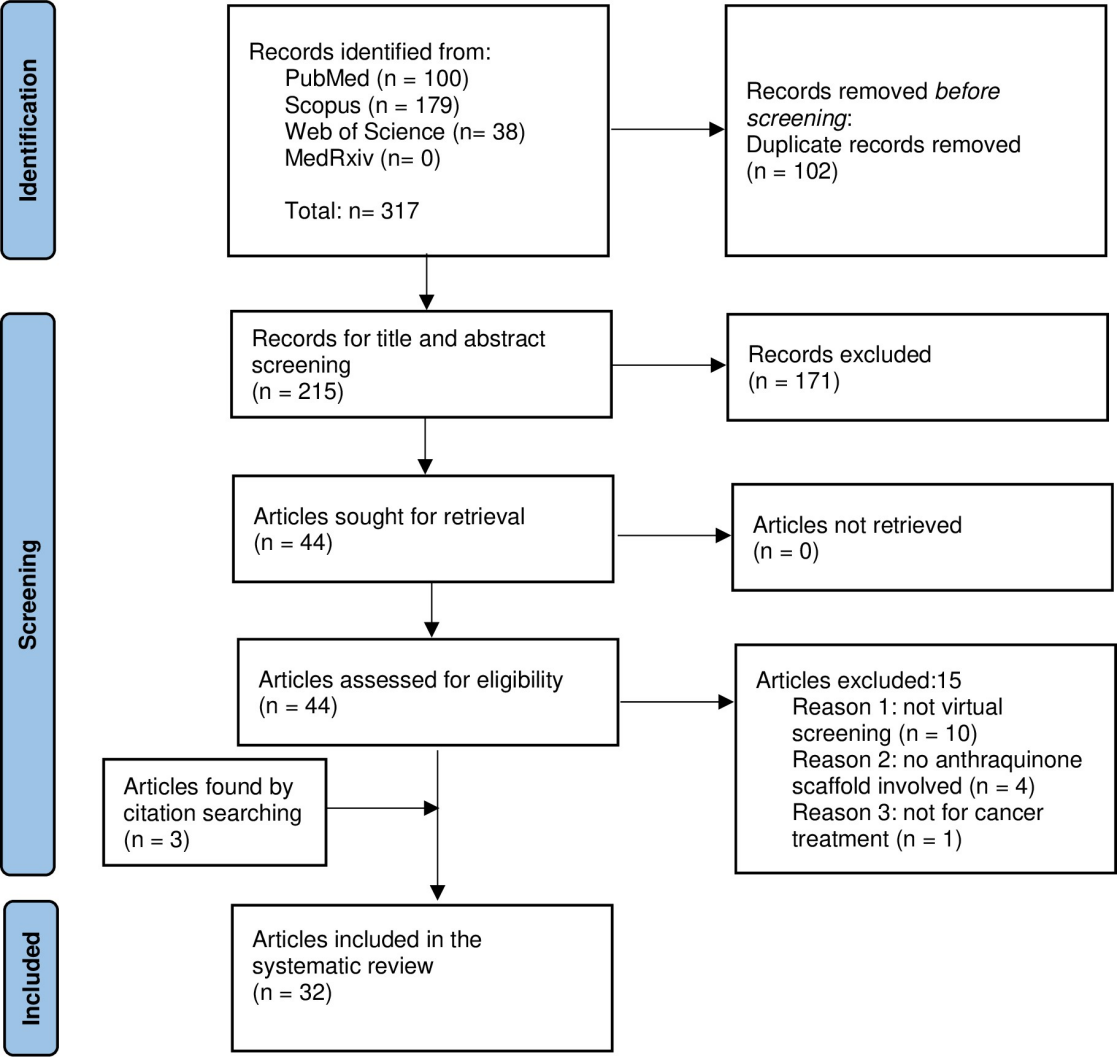
PRISMA flow diagram.

Results of the literature search from the databases were exported into the reference manager, Endnote X9.0 where duplicate publications were removed by following the steps as described by Bramer et al. [48]. After deduplication, two reviewers (HMC & MS) independently screened the title and abstract of the records to ascertain their relevance to the review questions. Afterward, the selected full-text articles were retrieved and read in detail by the same two reviewers according to the inclusion and exclusion criteria. The reasons for exclusion were recorded. Disagreements between the two reviewers were resolved through discussion with a third author (LM). The study selection process was recorded in the PRISMA flow diagram (refer to Fig 1 under Result and Discussion).

### Risk of bias assessment

Due to the lack of a standardised tool for this type of study, the risk of bias of the selected papers was assessed using a checklist previously developed and applied by Taldaev and colleagues [49], with some modifications. The assessment was carried out separately by two independent reviewers (HMC & MS). Disagreement was resolved by discussion with another reviewer (LM). This tool was mainly focused on the reporting quality of the molecular docking study. The original checklist consists of 7 main bias domains and 12 sub-domains.

For this review, the bias domain of “Docking Software” was removed. The authors who developed this tool ranked Glide or GOLD docking software as “low risk of bias” [49] since the Monte-Carlo algorithm and empirical scoring functions used by these two software have been shown to perform better as confirmed by in-vitro validation [50]. However, this was not always the case. Cheng et al. concluded that ‘none of the scoring functions works best at all time’ after assessing the “docking power”, “ranking power” and “scoring power” of 16 scoring functions implemented in popular commercial and academic software [51]. Another study compared five commercial and five academic docking programs in which GOLD and LeDock (both are commercial software) had the best sampling power whereas the academic software, AutoDock Vina was superior in terms of scoring power, implying commercial programs did not outperform academic software as expected [52]. More recently, Reddy et al. demonstrated that Glide gave consistent results in terms of docking conformation, ranking and scoring accuracy, but AutoDock ranked as the best scoring accuracy among all other tested software [53]. Therefore there is no single docking method that gives the best outcome for all docking jobs and the quality of docking result is greatly influenced by the ligands and target of interest [54].

On the other hand, the sub-domains of “Ligand optimization” were merged under “Ligand Preparation”. Likewise, “Target Optimization” was modified to “Target Preparation” and all relevant sub-domains were merged. As long as the ligands and target structures were prepared by using a special tool before undergoing docking calculation, the studies were ranked as “Low risk of bias”. This made up a total of 5 main bias domains and 9 sub-domains (Table 2).

**Table 2:** Risk of bias assessment.

Bias Domain	Sub-domain	Low Risk of Bias	High Risk of Bias	Unclear
Ligand selection	Ligand filtering	Performed	Not performed	No data
Ligand Preparation	Geometry-optimized and generation of energetically possible conformation	Performed by special tool	Not performed or performed without special tool	No data
Target Selection	Resolution of target structure	Not more than 2.5 Å	More than 2.5Å	No data
	Method of obtaining target structure	NMR spectroscopy	X-ray crystallography or cryogenic electron microscopy	No data
Target Preparation	Protonation, addition of missing residue and side chain after X-ray crystallography or cryogenic microscopy	Performed with special tool	Not performed or performed without special tool	No data
	Control of histidine and addition of metals	Performed	Target structure did not reference to biological condition	No data
Results assessment	Visual control	Performed	Not performed or structure defects observed	No data
	Redocking/ Docking Validation	Performed	Not performed or the RMSD value is higher than 2Å as compared to the initial structure	No data
	Verification of docking result by in-vitro study	Binding constant (eg: Ki, IC_50_) was determined	No laboratory validation or no quantitative calculations	No data

### Data extraction

Two independent reviewers extracted data from the eligible studies using a predefined data collection form (HMS & SM). The data extracted included the title of journal, authors, publication year, study context, CADD methods and the software/tools used, name and structure of the starting compounds or identified hits that contained anthraquinone scaffold, types of cancer involved, databases used to retrieve the structures of both target and ligand. (Refer S2 Table. Data collection form).

### Data synthesis and analysis

All included papers in the final studies were used for data synthesis and analysis. The data were summarized via a narrative approach to address our review questions. Tables and figures were used to present the characteristics of the studies. The trends and types of different CADD approaches and tools used in virtual screening as the primary tools for designing or discovering anthraquinone analogues for cancer treatment were analysed and discussed. The respective macromolecular targets involved and their role in managing different types of cancer, together with the chemical structures of the identified hit compounds that contained anthraquinone scaffold were presented and reviewed.

## Results and discussion

### Study selection

The literature search identified a total of 317 records. After importing the records into the reference manager (Endnote X9.0) where 102 records were deduplicated, 215 records were subjected to title and abstract screening by HMC and SM. The exclusion of 171 records that did not fulfil the inclusion criteria resulted in 44 records sought for full article retrieval. These articles were read in full and critically examined by the same two reviewers separately based on the eligibility criteria. The reasons for exclusion after full-text screening were recorded. Any disagreement was resolved by a third author (LM) through discussion and consensus. In addition, 3 more studies that fulfilled the inclusion criteria were found by citation searching, alongside the full screening process as they were somehow related to the included studies. The results of the study identification and selection process were recorded in the PRISMA flow diagram (Fig 1). A total of 32 articles were included in the final review.

### General view on the trend of publications

**Fig 2:**
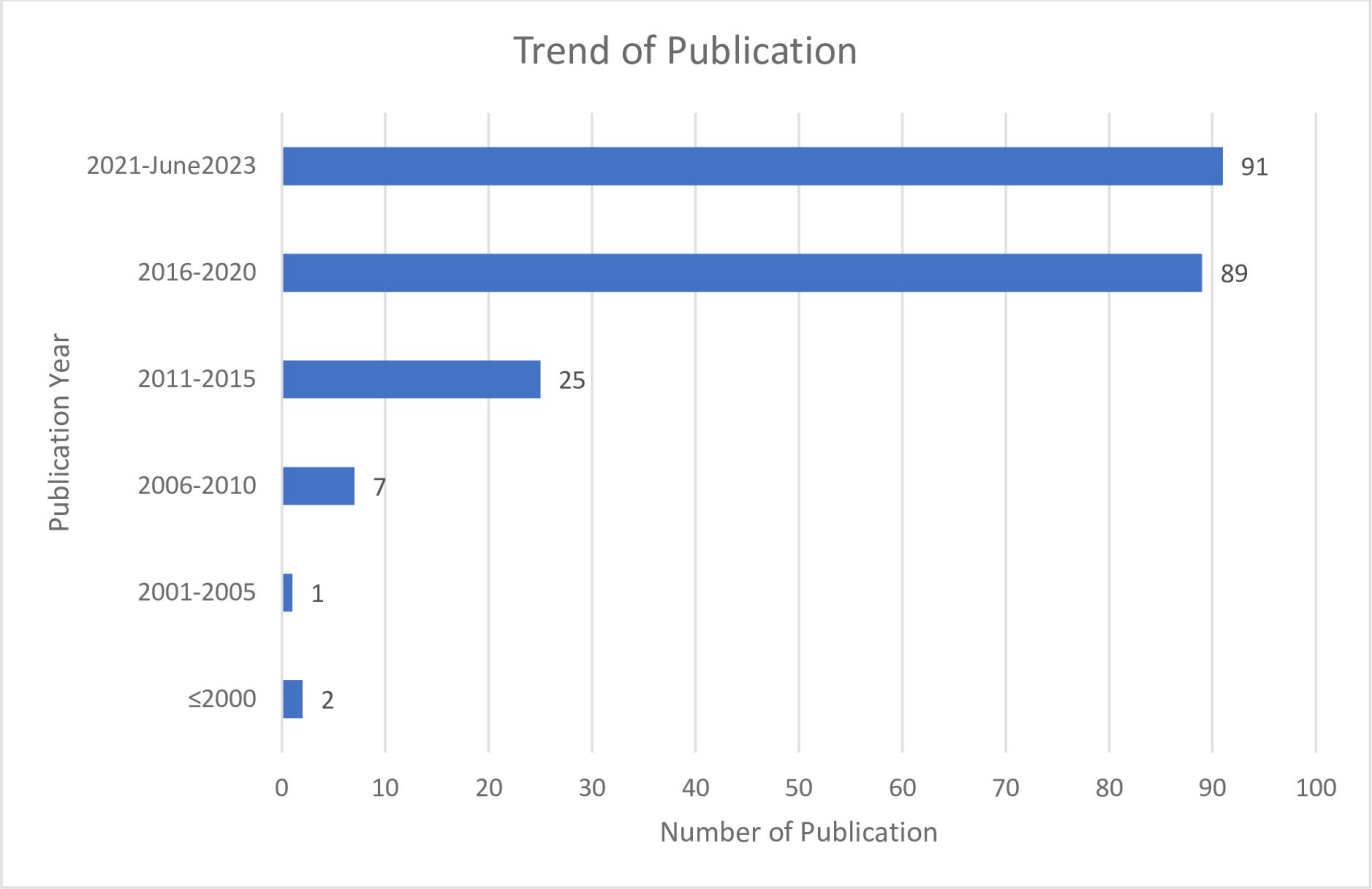
Trend of publications.

The number of publications in cancer research that involved CADD tools and anthraquinone was observed to be on the rising trend. From the total number of 215 articles retrieved from the search strategy after deduplication, only 2 articles were published before the twenty-first century. This number increased steadily after entering year 2006 (Fig 2). In fact, more than 90% of the identified articles were published in the last decade. From year 2021 up to June 2023 alone (less than 3 years), the highest number of publications (more than 40%) tagged with the searched keywords were recorded. This showed that the utilization of CADD methods has gained more popularity and anthraquinone is a compound of interest which attracted immense interest for biomedical research, especially in the oncology setting. One of the possible reasons may be also due to the worldwide lockdown caused by the Covid-19 pandemic in the past 3 years has switched many of the research focus from wet-lab to dry-lab (in-silico), hence more studies were performed virtually leading to more research publications on computer modelling. As virtual screening has been proven to offer an advantage in terms of resources, time and cost reduction in venturing novel drugs, it is expected that the number of research and publications in this field will continue to rise for the year 2023 and beyond.

### Types of CADD approaches used and the study context

Structure-based methods and ligand-based methods were used either independently or in combination. Table 3 illustrates the trends and different types of structure-based and ligand-based CADD methods used in the 32 included studies, together with their study context. Nearly one-third of the studies combined both structure-based and ligand-based methods in their drug design and discovery project (10 studies) while the rest used mainly the structure-based methods.

**Table 3:** Types of CADD methods used and the study context.

Authors (Year)	Structure-based methods	Ligand-based methods	Study Context	Reference
Ahire et al. (2016)	Docking	-	SBVS for hit identification	[55]
Arba et al. (2017)	Docking	-	SBVS for hit identification	[56]
Asnawi et al. (2022)	Docking	-	SBVS for hit identification	[57]
Bai et al. (2012)	Docking	Similarity search	SBVS + LBVS for hit identification	[58]
Ciaco et al. (2023)	Docking	Similarity search	LBVS + SBVS for hit identification	[59]
Choowong-komon et al. (2010)	Docking	-	SBVS for hit identification	[60]
Cozza et al. (2008)	Docking	-	SBVS for hit identification	[61]
Cozza et al. (2009)	Docking	-	SBVS for hit identification	[62]
Cozza et al. (2015)	Docking	-	SBVS for hit identification (re-evaluation)	[63]
Das et al. (2023)	Docking	-	SBVS for hit identification	[64]
DemiRezer et al. (2018)	Docking	-	SBVS for hit identification	[65]
Dong et al. (2019)	Docking	-	SBVS for drug repurposing	[66]
Gao et al. (2022)	Docking	-	SBVS for target verification and hit identification	[67]
Guan et al. (2020)	Docking	-	SBVS for drug repurposing	[68]
Golubovskaya et al. (2013)	Docking	-	SBVS for hit identification	[69]
Jordheim et al. (2013)	Docking	-	SBVS for hit identification	[70]
Khan et al. (2021)	Docking	Pharmacophore mapping	SBVS + LBVS for hit identification	[71]
Lankapalli et al. (2013)	Docking	-	SBVS for target fishing & hit identification	[72]
Leggett et al. (2022)	Docking	-	SBVS for hit identification	[73]
Liu et al. (2019)	Docking	-	SBVS for hit identification	[74]
Mhatre et al. (2017)	Docking	-	SBVS for hit identification	[75]
Nag et al. (2022)	Docking	Pharmacophore mapping	SBVS + LBVS for hit identification and poly-pharmacology study	[76]
Obounchoey et al. (2019)	Docking	Similarity search	LBVS + SBVS for hit identification	[77]
Rinne et al. (2020)	Docking, structure-based pharmacophore	Similarity search	SBVS + LBVS for hit identification	[78]
Roy et al. (2021)	Docking	-	SBVS for hit identification	[79]
Singh et al. (2021)	Docking	Scaffold hopping	LB-scaffold hopping SB-guided combinatorial library building & SBVS for hit identification	[80]
Song et al. (2019)	Docking	-	SBVS for hit identification & and hit-to-lead optimization	[81]
Taherkhani et al. (2021)	Docking	-	SBVS for hit identification	[82]
Wang et al. (2021)	Docking	3D-QSAR	LB + SB methods for novel drug design	[83]
Wu et al. (2018)	Docking	-	SBVS for Drug-repurposing	[84]
Wu et al. (2022)	Docking	Similarity search	LBVS+ SBVS for hit identification	[85]
Zyaater et al. (2019)	Docking	Similarity search	LBVS + SBVS for hit identification	[86]

None of the studies used ligand-based method alone since the crystal structure of all identified targets in the studies were either available in the protein databases [55, 56, 58–60, 62–67, 69–75, 77–86] or successfully created by homology modelling [57, 61, 68, 76].

The use of both structure-based and ligand-based methods were combined in either a parallel manner [58, 78, 80] or performed one after another in sequence, termed as sequential screening [59, 71, 76, 77, 83, 85, 86]. The sequential approach was more popular as it enabled efficient computation in which the more straightforward and quicker ligand-based virtual screening served as the pre-docking filter to reduce the size of the screening library before the more computational-demanding docking job took place. This is evident in those studies that successfully reduced the size of the screening database from 100 million of compounds in PubChem to merely 200 compounds [77] or from the Key Organic database of 49415 compounds to less than 10 compounds (85) after similarity search. Another study utilized ligand-based pharmacophore mapping from ZINC database with 20 million entries and identified only 12 phytochemicals analogues for docking [76]. Other studies that employed ligand-based pharmacophore or similarity screening prior to docking-based screening resulted in the shrinkage of library size that ranged from 30% to 85% [59, 86, 87].

On the other hand, running both approaches in parallel could enrich the hit discovery rate of virtual screening, and overcome the fundamental limitations of each approach [58] and this was particularly helpful when the sample size of experimental-validated known actives for use as references in virtual screen was limited [78]. For instance, Bai et al. designed a virtual screening protocol that started with a parallel screening of a selected database by molecular docking and shape-similarity search, followed by second stage docking in higher precision docking mode, then visually filtered out desired compounds for further bioassay, leading to a hit rate of 24.7% [58]. Rinne and colleagues ran five separate screening simultaneously (one by similarity search, two by pharmacophore mapping and two by molecular docking), each virtual screen shortlisted a hit list of 200 compounds for laboratory validation out of the database of 140000 compounds to tackle the limitation of virtual screen [78].

Majority of the studies aimed to discover novel hit or lead from the screened compounds or database, but three studies [66, 68, 84] looked into drug repurposing or repositioning from licensed medicines to explore additional indications in the oncology setting. The strategy of investigating new role of old drug offers benefits such as cheaper investment cost, shorter development time and reduced risk of failure since lesser efforts are required especially at the lead optimization stage [88].

There were three studies extended the exploration on the hit compounds identified from previous virtual screening studies. The starting compound, UM63 that contained anthraquinone scaffold used in one of the studies [59] to pre-filter the database based on similarity in SMARTS pattern was identified previously by the research group from the same university [86]. Similarly, the screening database of 200 compounds involved in another study [77] were defined based on Tanitomo coefficient of 0.95 from the eight hit compounds discovered by their colleagues previously. There was one study re-evaluated the selectivity of quinalizarin against CK2 by molecular docking and molecular dynamic simulation, and further measured the inhibitory effect of this compound on 14 kinases panels [63] compared to 7 kinases panels back in year 2009 when this hit was first shortlisted by the virtual screening study [62].

### Molecular docking

Molecular docking was the most popular structure-based method used in CADD. Notably, all of the 32 studies (100%) included in this review employed docking in their research (Table 3). This popular method is favoured due to its computational efficiency, in which the docking of one ligand on a single core typically requires only a few minutes of computing time [16]. Thus, docking-based virtual screening serves the purpose of filtering out good ideas from bad ones and subsequently eases the prioritization of more promising ligands out of the large size of the virtual library to be taken forward for subsequent studies.

Docking was also used in molecular modelling to investigate the interaction in between the shortlisted hits and the target protein in term of hydrogen bond, hydrophobicity, electron distribution and binding energy [55, 56, 63, 65, 75, 79, 82]. This was crucial to identify the structural determinants responsible for efficient binding with the amino acids or protein residues of the target. With this essential information in hands, promising drugs with more desired properties were designed [80, 81, 83].

One of the studies utilized molecular docking to verify the potential drug targets identified from the competitive endogenous RNA (ceRNA) network study by virtually screening out small molecule binders against these targets from the selected database [67]. On the other hand, molecular docking was also used in target fishing and profiling, in which the ligands were docked into a variety of macromolecules to predict appropriate targets for further analysis [72]. When one particular compound regulates multiple targets at the same time in the same illness, it is referred as “Polypharmacology” and again, molecular docking was useful to provide insights in this case [76].

#### Docking software

Docking methods consist of both posing and scoring process. Small molecules are docked into the macromolecular target to generate different ligand-target conformation and the degree of complementarity for each binding was ranked by scoring function. There were thirteen different docking tools used in the included studies in this review (Table 4). These were either commercial software with subscription fees or freely available (mainly for academic researchers). Each docking software has a different searching algorithm to align the binding geometrics of ligand-target complex to the preferred and stable pose. Different types of scoring functions are employed in these docking programs to evaluate the best binding pose of each bounded complex with minimum energy and rank the ligands accordingly from the most negative value to less negative value of the docking score.

**Table 4:** Summary of different docking tools used in the studies.

Docking Tool	Features	Website	Studies
Glide (Grid-Based Ligand Docking with Energetics)	Commercial software. Complete systematic search of the orientation, conformational and positional space of the ligand in the target with the OPLS-AA force field (Optimized Potentials for Liquid Simulations). Available in HTVS mode, SP mode, XP mode and induced fit docking.	https://www.schrodinger.com/products/glide	[58, 61, 62, 64, 68, 78, 80, 83]
GOLD (Genetic Optimisation for Ligand Docking)	Commercial software. Uses empirical score genetic algorithm for exploration of ligand flexibility. Examples of scoring function are GoldScore, ChemScore, Kinase Scoring Function (KCS).	http://www.ccdc.cam.ac.uk/	[60–63, 70, 77, 85]
AutoDock	Freeware. Uses Lamarckian genetic algorithm (LGA) posing where the conformations changes of ligands after optimization are used as subsequent poses for the offspring. The force-field-based scoring function considers the intermolecular interaction energy, the sum of torsional free energy, total internal energy, and unbound system energy.	https://autodock.scripps.edu/	[56, 57, 60, 66, 79, 81, 82]
AutoDock Vina	Freeware. A newer generation of AutoDock4. Uses knowledge-based scoring function and rapid gradient-optimization conformational search with Monte Carlo sampling technique.	https://vina.scripps.edu/	[67, 71, 75]
Surflex-Dock	Commercial software. A docking module in SYBYL software. Uses empirical scoring function by taking hydrophobic, polar, repulsive, entropic and solvated effects into consideration. The search engine is based on molecular similarity to dock ligands to target. Available in Normal, Screen, Geom and GenomX mode.	https://www.computabio.com/applications-of-surflex-dock-software.html	[65, 66, 68, 73]
FlexX	Commercial software. An incremental fragment-based docking algorithm where the conformational space sampling is done using a tree search method. Provided by BioSolveIT in the LeadIT package.	https://www.biosolveit.de/products/#FlexX	[55, 61, 62]
FRED	Free academic licensing program available. Uses exhaustive search algorithm that systematically searches conformers of each ligand within the active site at a specified resolution. Examples of scoring function are Chemgauss and Chemscore. Integrated in OpenEye Scientific Software.	https://www.eyesopen.com	[59, 86]
MOE-DOCK (Molecular Operating Environment)	Commercial software. A docking software under MOE Suite, an Integrated Computer-Aided Molecular Design Platform.	http://www.chemcomp.com	[61, 62]
UCSF DOCK	Free for non-commercial researchers only. Force-field based scoring.	https://dock.compbio.ucsf.edu/	[69]
PatchDock	Free for non-commercial researchers only. Surface path matching and molecular shape complementary algorithms followed by filtering and scoring.	http://bioinfo3d.cs.tau.ac.il/PatchDock/patchdock.html	[72]
iGEMDOCK (Generic Evolutionary Method for molecular DOCKing)	Free for non-commercial researchers only. An integrated graphical environment which utilizing post-screening analysis with pharmacological interactions for virtual screening.	http://gemdock.life.nctu.edu.tw/dock/igemdock.php	[75]
DockThor	Freeware. Uses MMFFLigand and PdbThorBox in-house tool for the docking algorithm along with MMFF94S53 force field.	https://www.dockthor.lncc.br/	[76]
Internal Coordinate Mechanics (ICM)-Pro	Commercial software. The molecular system was described by using internal coordinates as variables. Energy calculations were based on the ECEPP/3 force field with a distance-dependent dielectric constant. The biased probability Monte Carlo (BPMC) minimization procedure was used for global energy optimization.	https://www.molsoft.com/technology.html	[84]
PyRx virtual screen software	Commercial software. A virtual screening software that is using a variety of established open-source software including AutoDock4, AutoDock Vine, AutoDock Tools, Python, Visualization Toolkit, Open Babel etc.	https://pyrx.sourceforge.io/	[74, 89]

The most popular commercial docking software used in this review was Glide provided by Schrodinger, followed by GOLD provided by the Cambridge Crystallographic Data Centre (CCDC). For the academic software, AutoDock was among the most sought-after freeware that used by seven of the included studies. This freeware was developed by the Scripps Research Institute and they also have another newer generation of docking software namely AutoDock Vina. It was used by a total of five studies, where two of them performed the docking study using AutoDock Vina via the commercial virtual screening platform, PyRx (Table 4).

The popular docking programs normally have user-friendly interfaces with readily available integrated tools required for smooth handling of the in-silico workflow. For example, structure-rendering, visualization, target preparation (via either interactive or automated protein preparation workflow), ligand preparation (via LigPrep) could be all managed by the Maestro graphical interface developed by Schrodinger before submitting the Glide docking calculation job [58, 61, 62, 64, 68, 78, 80, 83].

### Consensus scoring

Some of the studies used two or more scoring functions to evaluate the best hit. This technique is known as ‘consensus scoring’ whereby different types of scoring functions are combined with the hope to compensate deficiencies of each scoring function and to improve capability of the screening process in discriminating actives from decoys [60–62, 66, 75]. The docking protocols of two studies were made up of four different algorithms (MOE-Dock, Glide, FlexX and Gold) and five different scoring functions (MOE-Score, Glide-Score, Gold-Score, Chem-Score and X-score), each was performed independently. The top-ranked compounds taken forward for further analysis were prioritized from the ‘consensus scored list’ generated from combining these docking programs [61, 62]. Another study used an empirical score genetic algorithm from the GOLD program and force-field genetic algorithm from AutoDock program (combined with FRED calculation) to rank the binding of ligands against the target protein, in which the highest scored compounds were also consensus between these two programs [60].

Two other studies utilized different precision modes of the selected docking software prior to re-docking by second docking software with varied scoring functions, aimed to improve the efficiency and accuracy of the virtual screening. One of them performed the initial virtual screening by Surflex-Dock in screen mode, followed by redocking of shortlisted hits with Surflex-Dock GeomX mode which featured higher spin density and accuracy. Meanwhile, the redocking was also conducted by AutoDock program that used a different scoring algorithm in parallel [66]. The other study combined Glide [Standard Precision (SP)], Extra Precision (XP), induced-fit docking and Surflex-Dock (Screen Mode, SurflexDock Geom and SurflexDock GeomX), in which each program was run by three different precision modes hierarchically [68]. Mhatre et al. also combined two different docking programs, namely iGEMDOCK and AutoDock Vina in their study but the operations were divided into two phases, iGEMDOCK was used in the first phase docking followed by redocking using AutoDock Vina in the second phase. The docking scores for each program were presented in a separate table and comparison was performed to analyse the preferred binding modes of selected ligands against the target. The results revealed that the pharmacophoric and molecular space acquired by the selected ligands were similar to the known active, implying the therapeutic potential of the phytochemicals of interest [75].

Apart from combining different scoring functions of various docking programs, the concept of consensus scoring was also applied in virtual screening that combined structure-based and ligand-based methods. One of the recent studies integrated docking-based virtual screening with three other ligand-based screening tools (pharmacophore, shape similarity and QSAR) in which each method produced separate hit lists and consensus Z-score for each highest-ranked ligand was then calculated [90]. This paper was published after the article searching period of this review ended therefore it is not included in the final review. However, the proposed strategy is worth further exploration.

### Pharmacophore modelling and mapping

In ligand-based pharmacophore screening, the 3D structures of a set of known actives were retrieved to guide the development of the pharmacophore model. This model served as the tool for subsequent virtual screening to map a predefined database for best-fit compounds that presented shared common features responsible for binding and biological functions [71, 76]. LigandScout was one of the commercial computer software used to generate the pharmacophore model and to score the compounds from the large database based on the computed pharmacophore features so that potential hits could be identified from the top-ranked list of the pharmacophore-fit score [71]. There was also a free web server, ZINCPharmar (http://zincpharmer.csb.pitt.edu/pharmer.html) equipped with ‘add feature’ function to predict pharmacophore features from the uploaded ligands candidates. ZINC database was then screened virtually by ZINCPharmer to pinpoint compounds that demonstrated the highest complementary to the model in terms of chemical descriptors such as hydrogen bonds, ring groups, ionic groups, hydrophobic and lipophilic groups [76].

There was also structure-based pharmacophore screening performed by Rinne and colleagues. Discovery Studio was used to develop the pharmacophore model. Maestro and Pymol from Schrodinger were used to aid the manual selection of pharmacophore features based on key residues at two binding cavities of the 3D target structure obtained from X-ray crystallography. This resulted in two separate hits lists from the pharmacophore-based screening [78].

### Similarity searching

Ligand-based similarity searching utilized the two-dimensional (2D) or three-dimensional (3D) descriptors of the known actives to discover most-alike molecules from the screening library based on the concept of ‘compounds with similar chemical structures tend to exhibit similar biological activities’ [58, 59, 77, 78, 85, 86]. Examples of 2D descriptors included molecular fingerprints [77, 78], substructure-based descriptors [85] or SMART patterns of the molecules [59, 86]. SMART refers to ‘SMILES arbitrary target specification’ whereby the full name of SMILES is ‘Simplified Molecular Input Line Entry System’. These are special languages developed by David Weininger and colleagues to describe structure or substructure patterns in molecules for cheminformatic purposes. FILTER application implemented in OMEGA from OpenEye Software was used to reduce the size of the screening database by performing SMART-based query prior to docking-based virtual screening [59, 86]. ChemBioFinder was another tool used to calculate the substructure search based on known active and to screen out potential candidates from the database with similar privileged motif as the query compound for further analysis [85]. Tanimoto similarity coefficient was a common metric used to rank the magnitude of similarity especially for molecular fingerprint mapping [77, 78].

Apart from structural information, 3D descriptors also take structural alignment into account for predicting the similarity between two compounds. Pharmacophore modelling and shape similarity are among the popular 3D methods for ligand-based virtual screening [91]. Bai and colleagues analysed the 3D conformers of co-crystalized ligands for similarity search using SHAFTS program and aided by the in-house conformational generation tool Cyndi [58]. SHAFTS (SHApeFeaTure Similarity) was developed to merge the pharmacophore overlay and shape complementary approach in discovering drug candidates with desired properties. Hybrid similarities score was calculated to prioritize the best-matched compounds with query compounds in terms of molecular pose alignment and volumetric superposition [92]. Obviously, 2D similarity approach was more popular as it was simpler, quicker and more straightforward. Nevertheless, both 2D and 3D similarity approaches were proven to increase the efficiency of virtual screening especially by downsizing the large library to become more ‘target-focused library’ for subsequent in-silico research.

### QSAR

QSAR involves mathematics calculation and statistics to model the correlation in between the molecular descriptors and biological activities [93]. A group of active compounds with the corresponding binding constant or inhibitory concentration determined by in-vitro studies was gathered as the starting point of the process flow. The active compounds were randomly divided into training sets and test sets in a predefined ratio. SYBYL software was used for molecular alignment based on the most potent compound to fix a common substructure. The popular CoMFA (Comparative Molecular Field Analysis) and CoMSIA (Comparative Molecular Similarity Indices Analysis) methods were then used to construct the 3D-QSAR model [83]. CoMFA is a force field based method that involves linear function but CoMSIA uses an exponential function to compute ligand properties such as steric and electrostatic energies [94]. The 3D-QSAR model was useful to guide lead optimization and new drug design by modifying the structure of existing ligands [83]. Both QSAR model and pharmacophore model are considered essential features of the known actives but the focus of QSAR model was more on features that correlated closely to the biological effect.

### Scaffold hopping

Scaffold hopping can be categorized as one of the ligand-based virtual screening methods as it typically requires the core structures of known actives to be used as the template [95, 96], at the same time it is also one of the main aims of many drug discovery projects to identify novel chemotype [91, 92]. Singh et al. modified the base scaffold of anthrafuran that was predetermined to demonstrate desired biological properties, guided by the binding cavity of the target of interest to build a combinatorial library of over 2 million new compounds based on in-silico enumeration. This target-focused virtual library was then subjected to structure-based virtual screening in which novel compounds with better biological activities than the parent analogue were discovered [80].

### Drug-likeness, lead-likeness and ADMET filter

ADMET properties are important for the ultimate fate of a possible drug candidate. Unwanted effects in animal models or even human trials can be reduced by filtering drug candidates by their drug-likeness, lead-likeness and ADMET properties in early stages [55]. These filters can be applied on the selected database prior to virtual screening [61, 69, 70, 76, 80] or afterward on the shortlisted hits [59, 64, 71, 75, 78, 81, 82], or even before and after [71].

The popular Lipinski’s Rule of Five [97] was applied to filter out compounds that disobeyed the drug-likeness properties as predicted (more than 5 hydrogen donors, more than 10 hydrogen acceptors, molecular weight larger than 500 and CLogP, a measurement of lipophilicity greater than 5) [55, 69–71, 75, 76, 80–82]. The freely accessible SwissADME server (http://www.swissadme.ch/) was another useful tool to predict properties like bioavailability, lipophilicity, pKa, blood-brain barrier permeabilities etc [64, 76]. The toxicity prediction technique included TOPKAT (Toxicity Prediction by Komputer-Assisted Technology) that applied QSTR (Quantitative Structure-Toxicity Relationship) models to deduce toxicity profiles such as carcinogenicity, mutagenicity, skin irritation was executed via Discovery Studio software package by Accelyrs [55]. PAINS (Pan-assay Interference Compounds) filter was also important to remove compounds with unwanted functional groups that might cause unexpected interactions with multiple targets leading to false positive results [59, 78].

Obviously, the studies involved drug repurposing could just skip the filtration step since the licensed small molecules are believed to possess desired physicochemical properties with acceptable bioavailability and safety aspects for oral consumption, as approved by the regulatory authority [66, 68, 84].

### Molecular dynamic simulation

The top-ranked protein-ligand complex shortlisted from docking studies were subjected to molecular dynamic simulations to observe how every atom in protein moved over time to assess the binding mode in depth, to confirm the stability of the docked pose and to gain insight on the protein flexibility of the ligand-protein complex [55–57, 63, 66, 76, 79, 80, 82, 83].

The most widely used software package to perform the simulation was AMBER [56, 66, 79, 83]. Throughout the years, different versions of the software package have been introduced, for instance AMBER12 [56], AMBER14 [66] and AMBER16 [79, 83]. Other molecular dynamic tools used were Discovery Studio Molecular Dynamic Protocol [55, 82], GROMAC [57], NAMD [63], DESMOND [80] and CABS-flex 2.0 server [76]. Among these tools, only DESMOND from Schrodinger required a license subscription fee whereas the rest were readily downloadable from the public domain for academic research purposes (Table 5).

**Table 5:** Molecular dynamic simulation software used.

Molecular dynamic simulation software	Brief description	Website	Used by
AMBER	Freeware	https://ambermd.org/	[56, 66, 79, 83]
Discovery Studio Molecular Dynamic Protocol	Freeware	https://discover.3ds.com	[55, 82]
GROMAC	Freeware	https://www.gromacs.org	[57]
NAMD	Freeware	https://www.ks.uiuc.edu/Research/namd/	[63]
CABS-flex 2.0 server	Freeware	http://biocomp.chem.uw.edu.pl/CABSflex2	[76]
DESMOND	Commercial software	https://www.schrodinger.com	[80]

All of the studies probed the dynamic of the protein-ligand complex in the time-scale of nanoseconds [55–57, 63, 66, 76, 79, 80, 82, 83]. Ideally, performing simulation in the order of microseconds or even milliseconds is better to reveal the biologically essential conformational evolution but unfortunately, this time-scale of simulation takes too long time to run and therefore rarely in use [54]. The stability of the ligand-protein complex was monitored by RMSD (Root Mean Square Deviation), a common metric used in evaluating the dynamic of macromolecules [55–57, 63, 66, 76, 79, 80, 82, 83]. The docked pose was considered stable if the docked ligand deviated from the macromolecules with an RMSD of less than 2 Angstrom over the simulated time range [57]. Some of the studies presented RMSF (Root Mean Square Fluctuation) to assess the protein flexibility of the ligand-protein complex [56, 76, 79].

### MM-GBSA/ MM-PBSA

There were seven studies predicted the binding free energy of the ligand-target complex using the molecular mechanics-Poisson-Boltzman solvent accessible surface area (MM-PBSA) [56, 57] or molecular mechanics-generalized Born surface area (MM-GBSA) methods [64, 66, 79, 80, 83]. These methods describe the binding free energy of a complex as the subtraction of the unbound macromolecule’s and unbound ligand’s free energy from the bounded macromolecule’s free energy [56]. Binding free energies of a complex also known as molecular mechanics potential energy, which is composed of the energy of both bonded (bond energy, angle energy, torsion energy) and non-bonded interactions (electrostatic energy, van der Waals energy) [56, 57].

A variety of different software packages are used for this calculation including the Python modules and NMODE module in AMBER [56, 66, 79, 83], PRIME [64, 80] and GROMAC [57]. All of the studies calculated the free energy of each system based on the snapshots taken from the molecular dynamic simulation trajectories except one study that did not perform the simulation beforehand [64].

### Source of crystal structure for macromolecular target

The availability of 3D structures of macromolecular targets is the prerequisite for performing structure-based virtual screening. More than 80% of the macromolecular structures used in the studies were retrieved from RCSB PDB (Research Collaboratory for Structural Bioinformatics Protein Data Bank). The crystal structures of the protein were either determined by either X-ray crystallography, NMR spectroscopy or cryogenic microscopy method, and deposited in the Protein Data Bank for free download in 3D format (https://www.rcsb.org/) [55, 56, 58–60, 62–66, 69–75, 77–86]. There was only one study obtained the 3D structure of the protein from AlphaFold, an artificial-intelligence based database that also provides free downloads of protein structures (https://alphafold.com) [67].

There were four studies that used homology modelling to construct the protein structures since those 3D structures were not readily available in the existing database [57, 61, 68, 76]. To start with, template searching was performed by SWISSMODEL, the well-known protein modelling online server (https://swissmodel.expasy.org/). Next, the amino sequence of the templates was downloaded from NCBI protein resources (https://www.ncbi.nlm.nih.gov/protein) followed by alignment of the target sequence and template structure, and finally the models were built by SWISSMODEL [57, 76]. Another modelling tool available for constructing homology modelling was the Molecular Operating Environment (MOE) [61, 68].

Validation of the model was also performed to ensure the quality of the modeled protein. Various online tools were available to assess and validate the model, namely WHATCHECK, PROCHECK, ERRAT, Verify3D, PROVE, OMEAN and Ramachandran plot [57, 61, 68, 76].

### Ligand databases

The databases used in virtual screening were either a public database, corporate collection, vendor database, a set of potentially synthesizable compounds or a combinatorial library built by in-silico enumeration of pre-defined core structures. Table 6 briefly describes various online ligand databases used in the included studies.

**Table 6:** Online ligand databases used.

Database	Brief description	Website	Used by
PubChem	Freely accessible database contains data on chemical structures, identifiers, chemical and physical properties, biological activities, toxicity data and many others. Launched in year 2004 by National Institute of Health.	https://pubchem.ncbi.nlm.nih.gov/	[55, 57, 71, 72, 75–77, 82]
ZINC	Freely accessible database contains purchasable compounds in ready-to-dock format. Provided by the Irwin and Shoichet Laboratories in the Department of Pharmaceutical Chemistry at the University of California, San Francisco (UCSF).	https://zinc.docking.org/	[70, 71, 73, 76, 81, 84]
DrugBank	Freely accessible database contains FDA approved small molecule drugs, FDA approved biotech drugs, withdrawn drugs, and experimental drugs. Started in 2006 by Dr David Wishart’s laboratory at the University of Alberta, Canada.	https://go.drugbank.com	[64, 66]
NCI/DTP database	Freely accessible database developed by National Cancer Institute (NCI), Developmental Therapeutic Program (DTP).	https://dtp.cancer.gov/	[60, 69]
MMsINC	Freely accessible database contains around 4 million of synthetic and natural compounds. Maintained by University of Padova, Italy	http://mms.dsfarm.unipd.it/MMsINC/search	[61]
Chemical library by University of Helsinki	A library of about 240,000 compounds maintained by University of Helsinki, Finland. Includes a subset of approved drugs, natural products and diversified sets of drug-like compounds.	https://www.helsinki.fi/en/infrastructures/drug-discovery-chemical-biology-and-screening/infrastructures/high-throughput-biomedicine/chemical-compound-libraries	[78]
FDA-approved library by Selleck	A unique collection of FDA-approved drugs and pharmacopoeia-included API.	https://www.selleckchem.com/screening/fda-approved-drug-library.html	[68]
Maybridge	Commercial database contains ready-to-screen library, fragment library, drug-like compounds library and few others.	http://www.maybridge.com (old) https://www.thermofisher.com/bn/en/home/global/forms/lab-solutions/maybridge-library.html (new)	[58]
SPECS database	Commercial database contains drug-like small molecules collected from academic sources worldwide.	https://www.specs.net/	[58]
Key Organic Database	Commercial database contains fragments and screening libraries, building block libraries etc.	https://www.keyorganics.net/	[85]
Aldrich Market Select	Commercial database by Sigma Aldrich, USA.	https://www.aldrichmarketselect.com/	[59]
MolPort	Commercial database. Launched in Riga, Latvia.	https://www.molport.com/	[59, 86]
Herb database	Traditional Chinese Medicine database guided by references and high-throughput experiments. Created by researchers in Beijing University of Chinese Medicine.	http://herb.ac.cn/	[67]
TCM Database@Taiwan	Freely accessible small molecules database on Traditional Chinese Medicine. Constructed by Computational Systems Biology Laboratory at the China Medical University (Taiwan).http://researcher.nsc.gov.tw/ycc0929/	http://tcm.cmu.edu.tw	[74]
Plant Database	Contains phytochemicals of medicinal plants from East Africa, North Africa, North East	Not found	[64]
Molecular Modelling Section (MMS) Database	In-house database by Molecular Modelling Section (MMS), Department of Pharmaceutical Sciences, University of Padova, Italy	Not found	[62, 63]

PubChem and ZINC were among the most popular databases used. Both were public databases with more than millions of chemical structures readily downloadable in several common file formats. As of today, PubChem contains 110 million compounds whereas ZINC20 (the current version) contains 750 million molecules with 230 million purchasable compounds in ready-to-dock format (both websites accessed on 23 October 2023).

The size of these popular databases has grown in an exponential manner throughout the years. When Leggett et al. performed their virtual screening, ‘2007 ZINC drug-like library’ was used whereby the number of molecules contained was reported at about 700 hundred thousand [73]. The reported number was increased to 120 million when another study screened the database (ZINC15) about a decade later (81). ZINC database also contains different subsets to tailor for different aims of screening. For example, the Acros Organic database used by Jordheim et al. was one of the subsets after filtering with the Lipinski rules [70]. On the other hand, Khan and colleagues used the subset of synthesizable natural compounds (Zbc) loaded with biogenic lead-like compounds, primary and secondary metabolites of natural products [71]. Last but not least, the database of ‘FDA-approved drugs (via DSSTOX)’ used in another study was also obtained from ZINC (http://zinc.docking.org/catalogs/fda) [84].

It is inevitable that some of the compounds are overlapping in various libraries, but each library still carries unique features. In this review, some of the studies used more than one database to cater to their different objectives, these included a combination of two public databases [71, 76], two commercial databases [58, 59], or a plant database together with a synthetic database [64]. In order to optimize the screening outcome, another useful strategy was the construction of a virtual combinatorial library by enumerating the main scaffold of existing active compounds with a pre-selected drug block in which a target-focus library could be formed [80].

It is also worth highlighting the efforts of the research community in building natural product databases owing to the increasing momentum seen in herbal research. The HERB database used by Gao et al. [67] was built by linking targets and diseases to herbs, aiming to aid the modernization of Traditional Chinese Medicine especially in rational drug design [98]. Besides, another TCM database@Taiwan used in one of the studies [74] was designed in both English and Chinese languages, deemed as the largest collection of 3D structures for Traditional Chinese Medicine to date in a freely accessible ready-to-dock format to support the in-silico research [99].

Ligand constructors were used to draw the 3D structures of the ligands when the data required was not readily available. Among the tools used included Chemicalize [55], GaussView [56], SYBYL sketcher module [65], ACD/Chemsketch [75], Marvin Sketch [80], and ChemDraw [83].

### Identified targets and anticancer properties of anthraquinone derivatives

**Fig 3:**
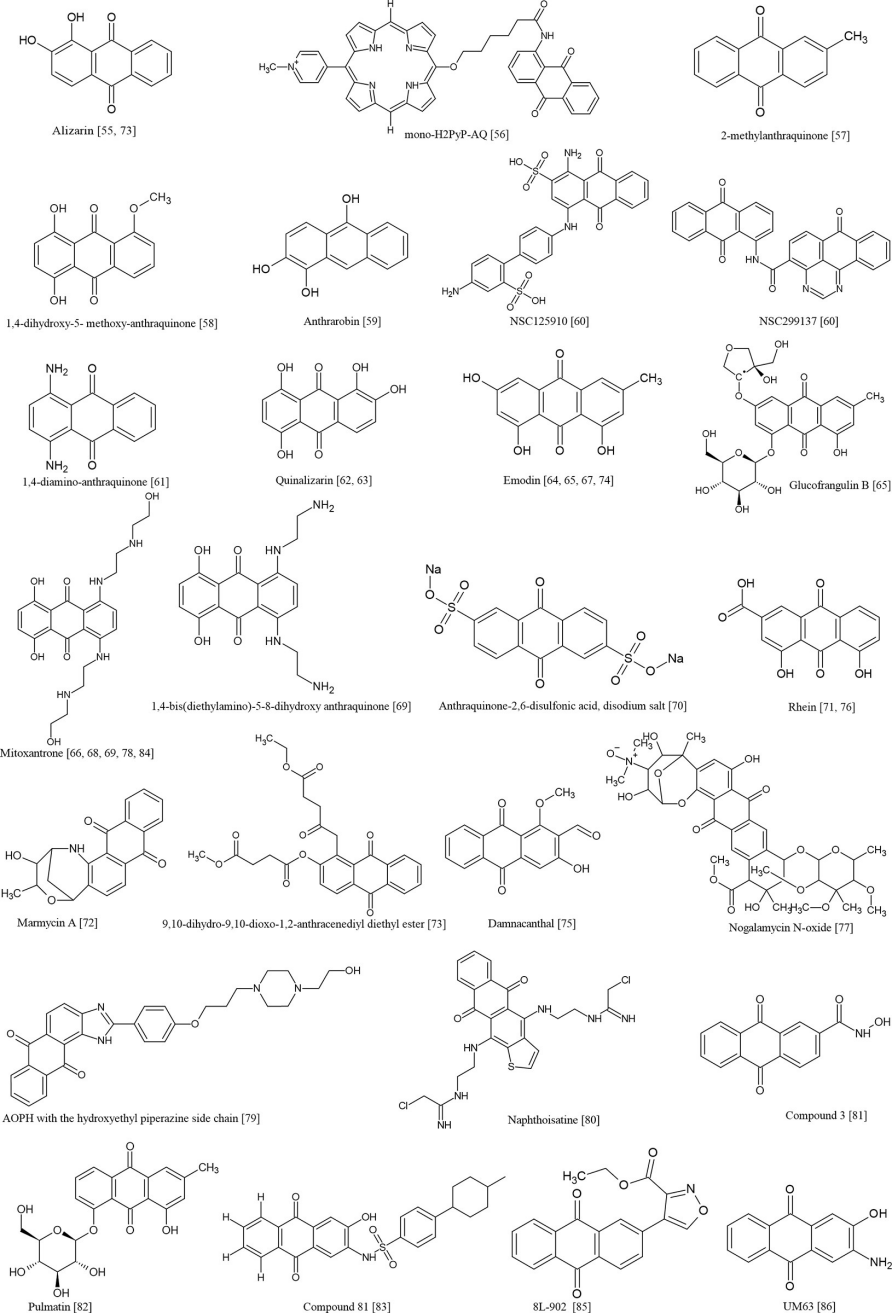
Chemical structure depiction of the hit compounds.

The main target proteins investigated in the included studies for developing potential therapeutics against different types of cancer are presented. The best hit was either derived from a set of starting compounds that contained anthraquinone scaffold or discovered by screening varied sizes of databases. The identified hit compounds that contained anthraquinone scaffold, the observed interactions with the target proteins simulated by the molecular modelling and their corresponding potency (if tested experimentally) are also displayed (Table 7). (Refer to Fig 3 for the chemical structure depiction of the respective hit compounds).

**Table 7:** Target proteins and anticancer properties of anthraquinone derivatives elucidated by CADD methods.

Database/ Compounds Screened	Target(s) Involved: PDB ID	Cancer Type	Name & Structure of Identified Best Hit Derived from Anthraquinone	Observed Interactions/ Predicted Binding Mode by the Molecular Modelling	In-vitro Validation (Potency)	Study
Structural derivatives of 1-hydroxy-2-methyl-anthraquinone retrieved from PubChem	p53 Y220C mutant: 1TUP and 2XOW	General	Alizarin (Among the best six ligands)	Not reported	Not performed	[55]
Six cationic porphyrin-anthraquinone hybrids built with the help of GaussView	Cyclin-Dependent Kinases (CDKs): 1DI8	General	Porphyrin-anthraquinone hybrid with one substituent (mono-H2PyP-AQ)	Anthraquinone tail projected to the hydrophobic region while the porphyrin core interacted with the hinge residues Phe80, Glu81, and Leu83	Not performed	[56]
Sixty phytochemicals of *Acalypha indica* retrieved from PubChem	BRAF kinase: 6XFP (homology modelling template)	Melanoma	2-methyl anthraquinone (Among the top four phytochemicals)	Hydrophobic interactions were observed with Ile463, Val471, Ala481, Trp531 and Phe583.	Not performed	[57]
Vendor Database (Maybridge and SPECS)	Epidermal growth factor receptor (EGFR) wild type (WT) and EGFR T790M mutant: 2JIV, 2JIU and 3IKA	Lung Cancer	1,4-dihydroxy-5- methoxy-anthraquinone (Compound 4)	Hydrogen bonds observed with Met793, Gln791 and a conserved water molecule via two hydroxyl groups and a methoxyl group.	IC_50_ EGFR-WT: 4.3μM; IC_50_ EGFR-T790M/ L858R: 17.6μM	[58]
UM63 as starting compound for similarity search in MolPort (7,591,844 entries) and Aldrich Market Select (8,512,248 entries)	Ubiquitin-like containing PHD and RING fingers domains 1 (UHRF1): 3CLZ	General	Anthrarobin (AMSA2) -structurally related to UM63 with anthraquinone core	The isolated hydroxyl group H-bonded with the backbone of Val44 while the catechol moiety H-bonded with the backbone of Ala463 and Thr479, and the side chain of Asp469.	IC_50_ UHRF1: 5.40μM	[59]
National Cancer Institute (NCI) diversity database (1990 compounds)	Epidermal growth factor receptor (EGFR): 1M17	Lung cancer	NSC125910 (Compound G)	Compound G formed hydrogen bond with Lys721 via O-atom of the dioxoanthracene group, while the O-atom and H-atom of the sulfophenyl group H-bonded to the Cys773 and Asp776.	Not performed	[60]
			NSC299137 (Compound J)	Compound J formed a weak hydrogen bond between the backbone-N of Met769 and the carbonyl oxygen of the dioxoanthracene-ring.		
MMsINC (in-house database contained around 4 million of synthetic and natural compounds)	Protein Kinase CK1 delta (CK1d): 2CSN and 1EH4 (homology modelling template)	General	1,4-diamino-anthraquinone (Compound 1)	Stabilizing interaction was made between one of the amino groups and Glu83 while another amino group H-bonded with Asp149. Another hydrogen bond was observed with Leu85. Hydrophobic bonds were also observed with Ile15, Ile23, Ala36, Leu135, Ile147.	IC_50_ CK1d: 0.33μM	[61]
Molecular Modelling Section (MMS) database (more than 3000 of both synthetic and natural compounds)	Protein kinase CK2: 1JWH	General	Quinalizarin (1,2,5,8-tetrahydroxy-anthraquinone)	Not reported	IC_50_ CK2: 0.15μM	[62]
Quinalizarin (Identified from previous SBVS study)	Protein kinase CK2: 4MD& (CK2 apo form) and 3QA0 (CK2α apo form)	General	Quinalizarin (1,2,5,8-tetrahydroxy-anthraquinone)	Hydrogen bond formed between one of the hydroxyl groups with Val116 in the hinge region via a water molecule; another hydroxyl group H-bonded with His160 on one side, and with carbonyl of Arg47 on the other side.	IC_50_ CK2α_2_β_2_: 0.15μM; IC_50_ CK2α: 1.35μM	[63]
14000 phytochemicals from Plant Database and 14500 synthetic drugs from DrugBank	Maternal Embryonic Leucine Zipper Kinase (MELK): 5IH9	Breast cancer (especially Triple-Negative Breast Cancer)	Emodin	Hydrogen bond was formed with Cys89	IC_50_ TNBC cell line: 30.30μM	[64]
Five anthranoid skeletons and derivatives as hydroxy-anthraquinones constructed by the SYBYL sketcher module	Estrogen receptor alpha (ERα): 1A52	Hormone-associated diseases including cancer	Glucofrangulin B	Hydrogen bond interactions were observed with residues Glu423, Lys520, His516, His516, Arg515, Arg515, Arg515 and Cys381.	Not performed	[65]
	Estrogen receptor beta (ERβ): 1QKM		Emodin	Hydrogen bond interaction was observed with Glu305.		
DrugBank (contained 7097 compounds)	human O-GlcNAcase (hOGA): 5M7T	General	Mitoxantrone	The anthraquinone moiety pi-pi stacked with Phe223, Tyr286, and Trp645, and the hydroxyl groups on the anthraquinone ring H-bonded to Lys98 and Asp175. One of the hydroxyethyl side chains H-bonded with Lys98 and Asn313. The other side chain interacted with Lys648 outside the pocket.	IC_50_ hOGA: 7.3μM	[66]
HERB database for small molecules	Reticulo-calbin2 (RCN2): From AlphaFold database	Cholangio-carcinoma (CCA)	Emodin	Hydrogen bonds were observed with residues Gln261, Asn259, Tyr239 and Arg234.	Not performed	[67]
FDA-approved Drug Library (by Selleck, contained 1375 drugs)	eEF-2K (Eukaryo-tic elongation factor-2 kinase): 3LKM (homology modelling template)	Breast cancer	Mitoxantrone	Hydrogen bonds were observed with residues Arg140, Lys170, Ile232, Glu 233, and Gly234.	Kd to eEF-2K: 9.11 μM	[68]
NCI/DTP Small Molecules Database (contained more than 140,000 compounds)	Focal Adhesion Kinase (FAK): 2J0J and 2J0L	General	Mitoxantrone and derivative (A18 compound): 1,4-bis (diethylamino)-5-8-dihydroxy anthraquinone	A18 compounds docked into the K454 site of the FAK kinase domain	Not calculated	[69]
Acros Organics subset of the Zinc database (contained 13,754 compounds after filtering)	Human cytoplasmic nucleotidase (cN-II): 2JC9	General (especially acute myeloid leukemia)	Anthraquinone-2,6-disulfonic acid, disodium salt (AdiS)	Not reported	IC_50_ RL (lymphoma) cell line: 750 μM	[70]
-166 known inhibitors retrieved from PubChem (filtered for pharmacophore modelling)-Zbc library (26432 natural compounds)	ATP-binding cassette Super-family G member 2 protein (ABCG2): 5NJ3	General	Rhein (ZINC4098704)	Twenty-seven hydrophobic bonds were observed with residues Tyr613(2), Ile423(4), Gln424(5), Ser420(4), Lys616(3), Tyr605(2), Ala606(2) and Thr607(5). Four hydrogen bonds were observed with Gln424, Thr607 and Lys616.	Not performed	[71]
9 compounds extracted from marine *Streptomyces*, retrieved from PubChem	human epidermal growth factor receptor 2 (HER2): 1N8Z	General	Marmycin A (marine-sourced anthraquinone-derivatives)	Interactions were observed with residues Cys312, Pro315, Cys316, Arg318, Val319, Cys320, Asn297, Gln298, Glu299, Cys312, Pro315, Cys316, Arg318, Glu326, Met324, Tyr321, and Phe349.	Not performed	[72]
2007 Zinc drug like library (contained 2 million compounds)	Arylamine N-acetyl-transferase 1 (NAT1): 2PQT and 2PFR	General	9,10-dihydro-9,10-dioxo-1,2-anthracenediyl diethyl ester (Compound 10)	Not reported	IC_50_ NAT1:0.75 μM	[73]
			Alizarin		IC_50_ NAT1 0.89 μM	
TCM database@ Taiwan	G-quadruplex (G4s): 1KF1 (parallel) 143D (anti-parallel) 2JPZ (hybridized)	General	Emodin	Emodin docked at the binding site of 143D in a larger groove than in 2JPZ, and the hydroxyl of emodin H-bonded to the phosphoric acid oxygen atom of DT9. No ligands were observed for parallel G4 due to the lack of a pocket site on the surface.	Not calculated	[74]
Anthraquinones derivatives from *Morinda citrifolia* attained from PubChem	Dihydro-folate reductase (DHFR): 1DLS	General	Damnacanthal	Hydrogen bonds were observed with Asp21, Ser59, and Asn64. Van der waal interactions were observed with Tyr22, Phe31, Ile60.	Not performed	[75]
Four phytochemicals from *Amomum subulatum*, retrieved from PubChem for pharmacophore screening via ZINCPharmer	HPVE6:4GIZ; BCL2:2XA0; XIAP:1F9X; HPVE7 & LIVIN: homology modelling	Cervical cancer	Rhein (among the 4 phytochemicals identified)	Not reported	Not performed	[76]
200 compounds resulted from similarity search on PubChem based on eight starting compounds identified by previous study	Epidermal growth factor receptor (EGFR): 1M17	Lung cancer	Nogalamycin N‐oxide (NSC116555),	Binding interaction was observed with Thr854 in the DFG‐in conformation. Additional conserved hydrogen bonds were observed with Cys797, Ala743, Lys745, Asp855, Cys797, Leu844, and Phe856.	IC_50_ EGFR: 31.56 nM	[77]
Chemical Library of about 140 000 compounds maintained by University of Helsinki	Toll-like receptor 4 (TLR4): 3FXI	General	Mitoxantrone (Structure as above)	Not reported	Not calculated	[78]
25 Derivatives of Monomeric anthraquinone based, bis-benzimidazole and hybrid carbazole-benzimidazole type ligands	G-quadruplex (G4) RNA: 2KBP	General	AOPH with the hydroxyethyl piperazine side chain (The best among nine anthraquinone-based ligands)	The anthraquinone moiety partially stacked on the G-tetrad surface interacted with G17 and G2, and the hanging piperazine part interacted with the groove containing U18 and A20 of strand B.	Not performed	[79]
Combinatorial library of over 2 million compounds created from in-silico modification of anthrafuran	human aurora kinase B (AurB): 4AF3	General	Naphthoisatine (Compound 2)	Hydrophobic interactions and hydrogen bonds were observed with residues Leu83, Gly84, Lys85, Phe88, Tyr156, Ala157, Arg159, Gly160, Glu161, Lys164, Glu 165, Glu204 and Leu207.	IC_50_ AurB 7.4 μM	[80]
235 compounds that have ring subsets from ZINC15 database after filtered off macrocyclic molecules	Histone deacetyl-lases isoform 6 (HDAC6): 5EF7 and 5EDU	General	Compound 3	Hydrogen bonds were observed with the side chain of Y745, backbone amide oxygen of G582, and imidazole side chain of H573. The middle quinone and phenyl rings were sandwiched between lipophilic side chains of F583 and F643, forming pi-pi interactions. Two carbonyl oxygens of the quinone ring formed hydrogen bond with H614 and S531.	IC_50_ HDAC6: 56 nM	[81]
21 natural anthraquinone derivatives retrieved from PubChem	Matrix metallo-proteinase-13 (MMP-13): 5B5O	General	Pulmatin	Four hydrogens and four hydrophobic interactions formed with Gly183, Leu184, Ala186, Glu223, Ile243 and Tyr244.	Not performed	[82]
78 anthraquinone-based inhibitors retrieved from literatures used for 3D QSAR modelling	Phospho-glycerate mutase 1 (PGAM1): 5Y35	General	Compound Number 81 (among the seven new inhibitors designed based on the QSAR modelling)	Not reported	Not performed	[83]
3000 FDA-approved drugs from ZINC database	NEDD8-activating enzyme (NAE): 3GZN	General	Mitoxantrone	Three hydrogen bonds were observed with Thr103, Gln112, and Lys307 at the binding site. One of the alkyl chains extended out into the solvent region, while the other probed deeper into the binding pocket.	EC_50_ Caco-2 cell: 1.3 μM	[84]
Emodin analogues queried by substructure search from Key Organic database (49,415 compounds)	Aurora Kinase A (AURKA): 5ORL	Ovarian cancer	8L-902 (among the 9 analogues identified)	One hydrogen bond interaction, two pi–pi interactions, one pi–sigma interaction, and four pi–alkyl interactions were observed.	Not calculated	[85]
MolPort (6,504,839 molecules)	Ubiquitin-like containing PHD & RING fingers domains 1 (UHRF1): 3CLZ	General	UM63	Interaction was stabilized by pi-pi stacking with the side chain of Tyr478 and several hydrogen bonds to key residues Asp469, Thr479, Gly448, Gly465, Ala463.	IC_50_ to SRA-induced base flipping: 4.4 μM	[86]

Anthraquinone scaffold is a privileged scaffold that carries biological activities against a wide range of macromolecular targets. Privileged scaffold is defined as the core structure that can interact with more than one receptor with high affinity [100]. The prioritized anthraquinone derivatives from the included studies exhibit different types of interactions with the identified targets, these included hydrogen bonding, hydrophobic interactions, pi-pi stacking, pi-sigma and pi-alkyl interactions (Table 7). The calculated IC_50_ (half maximal inhibitory concentration) of these hit compounds determined from the in-vitro experiment ranged from micromolar [58, 59, 61–64, 66, 70, 73, 80, 86] to nanomolar [77, 81] scale, indicating the high affinity of these anthraquinone derivatives against the various macromolecule targets. There was one study reported the potency of the identified hit in the form of Kd [dissociation constant] [68] and another study calculated EC_50_ (half-maximal effective concentration) [84], both of these values were also in the micromolar scale, suggesting the potential of these hit compounds as the promising candidates for further development into new anticancer drugs.

**Fig 4:**
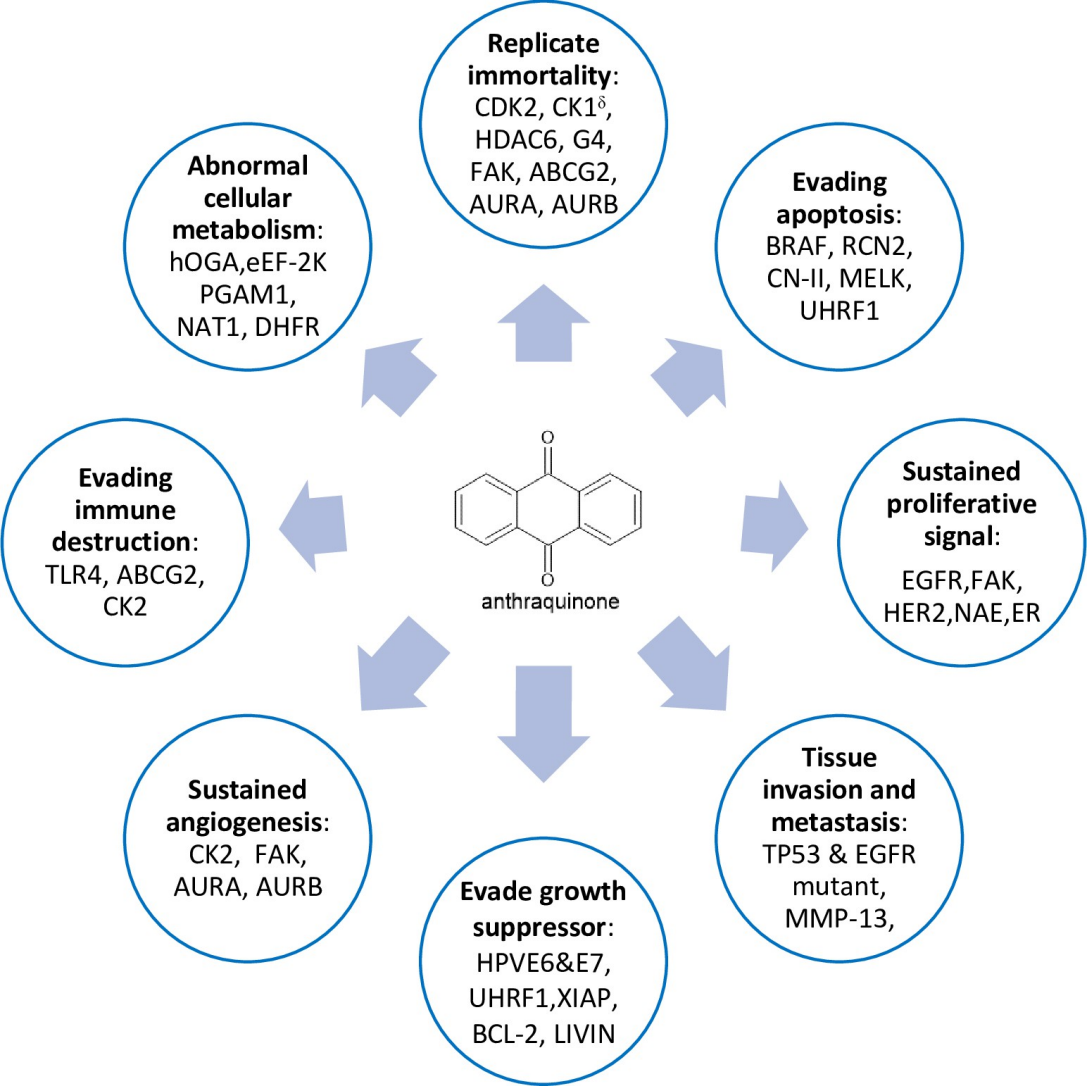
Targets involved that tackled the hallmarks of cancer.

The majority of the studies looked into the role of these targets in general cancer whereas some of them dived into specific cancer types. Among these were the top killer cancers in men and women namely lung cancer [58, 60, 77] and breast cancer [64, 68] respectively. Another two types of cancer that threaten the female population included cervical [76] and ovarian cancer [85]. Stubborn diseases with poor prognosis such as melanoma [57], cholangiocarcinoma [67] and acute myeloid leukaemia [70] were also investigated. The macromolecular targets involved are responsible for managing these different malignancies via various mechanisms, mainly to tackle the hallmarks of cancer [101] (Fig 4).

Many of the studies investigated the role of anthraquinone derivatives against the protein kinases family, the enzymes that carry multiple roles at once. The serine-threonine kinases like CDK2 [56], CK1δ [61], CK2 [62, 63], MELK [64], AURA [85], AURB [80], BRAF [57] involved in many cellular processes including signal transduction and controlling cell cycle progression in various phases. Another tyrosine kinase family investigated were FAK [69], EGFR [58, 60, 77], and HER2 [72] that mediated cell proliferation and survival. Dysregulation of these kinases in malignant cells resulted in a sustained supply of blood to the tumours, uncontrolled growth and immortality of the cells, in which anthraquinone derivatives managed to serve as inhibitors against these targets to activate apoptosis. Inhibition of targets like RCN2 [67] and cN-II [70] also induced programmed cell death by activation of natural killer cells.

Another calcium/calmodulin-dependent kinase known as eEF-2K stimulated the cancer growth by mediating through autophagy and facilitating the switch from oxidative phosphorylation to glycolysis in response to metabolic stresses [68]. Other cancer-promoting enzymes that involved in carcinogen metabolism included hOGA [66], PGAM1 [83], NAT1 [73] and DHFR [75]. Anthraquinone derivatives such as mitoxantrone, alizarin and damnacanthal managed to turn off this special metabolic pathway and increase the susceptibility of cancer cells to apoptosis induced by chemotherapy.

Apart from that, anthraquinone derivatives were also found to trigger telomere dysfunction by targeting G4 and suppressing tumour growth [74, 79]. Emodin, one of the naturally-occurring anthraquinones was reported to work synergistically with telomerase inhibitor in sustaining the telomere defect and enhancing cancer cell damage [74]. On the other hand, oncoproteins such as HPVE6, HPVE7 [76], UHRF1 [59, 86] and anti-apoptotic protein BCL-2, XIAP, LIVIN [76] are involved in protecting the cancer cells from growth suppressor and hence facilitating their growth. Anthrarobin and rhein were among the potential hits found to modulate these target proteins leading to cancer cells suppression.

Over-expression of proteins like mutated TP53 [55], mutated EGFR [58] and MMP-13 [82] enhanced the ability of cancer cells to evade adjacent tissues leading to metastasis. Alizarin, Pulmatin and 1,4-dihydroxy-5-methoxy-anthraquinone were identified as potential inhibitors for mutated TP53, MMP-13 and mutated EGFR respectively and help in preventing the spread of cancer cells. Another investigated target, TLR4 was found to serve as a mediator of innate immune system activation [78]. In addition, the elevated level of ABCG2 on the plasma membrane reduced the effect of anti-cancer drugs by enhancing the efflux process leading to multidrug resistance (MDR) [71]. This phenomenon was also observed with overexpression of CK2 [62, 63]. Virtual screening discovered quinalizarin, rhein and mitoxantrone as modulators of these targets and exhibit therapeutic roles in managing multidrug resistance. Last but not least, one of the studies showed that phytochemicals like emodin and glucofrangulin B also carried phytoestrogenic activities in which their effect on estrogen receptors was investigated [65]. These anthraquinone derivatives may play an important role in hormone-related disorders including breast cancer that commonly associated with elevated levels of estrogen in blood.

Taken altogether, this review shows that anthraquinone was proven to be a valuable compound containing the most privileged scaffold with great therapeutic potential to be developed into wide-spectrum anticancer drugs.

### Risk of bias assessment

**Fig 5:**
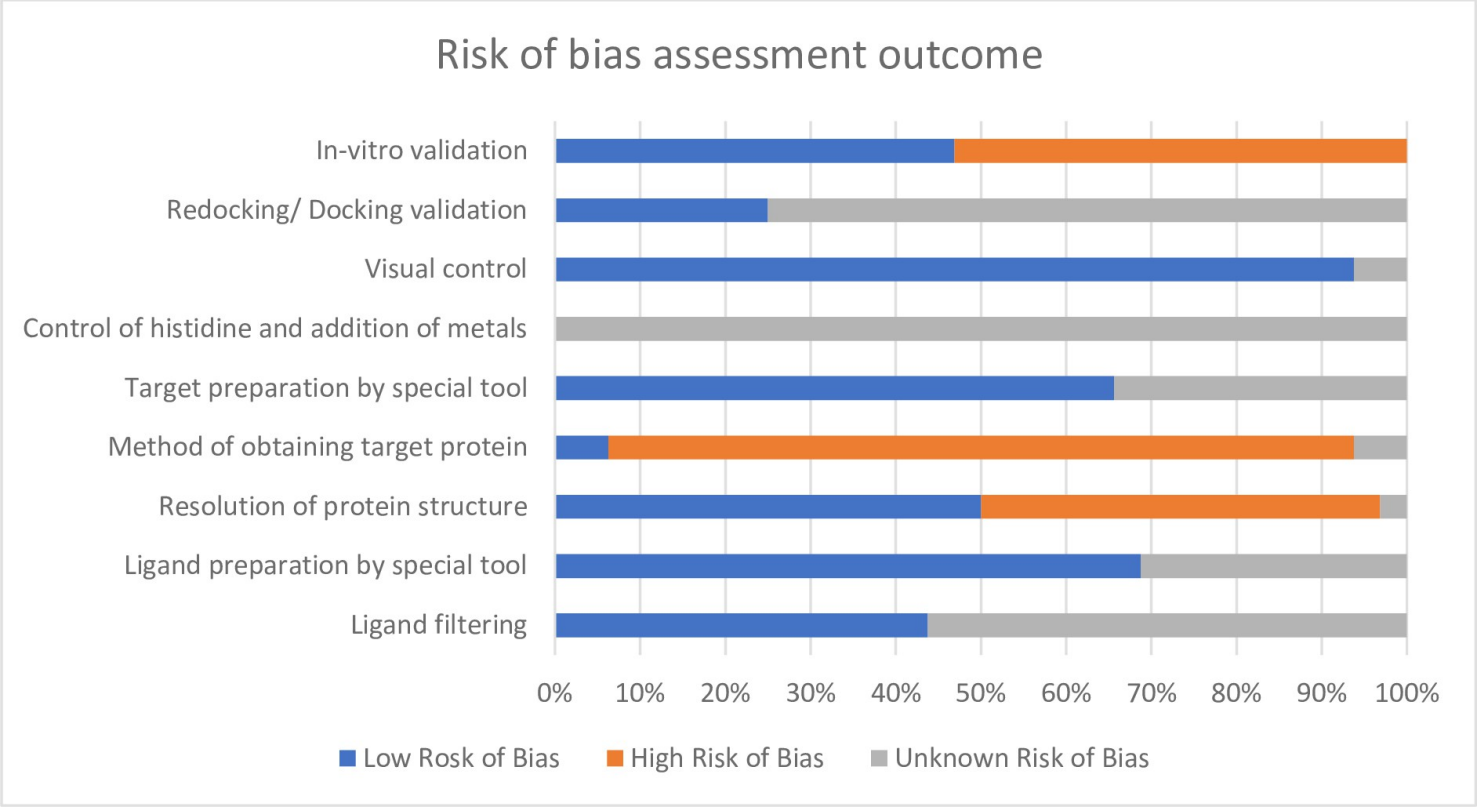
Risk of bias assessment outcome.

The risk of bias checklist was designed mainly to assess the reporting quality of docking-based studies (Table 2). Since molecular docking was involved as the main screening tool in all of the studies included in this review, the risk assessment managed to cover all papers. The results are illustrated in Fig 5.

For all 32 papers, none of them described on the control of histidine and addition of metal as part of their target preparation steps. It may be due to the lacking of clear guidelines to guide the proper reporting of in-silico research. Nevertheless, nearly 70% of the studies reported on the ligand optimization steps which include ionization assessment and generation of possible conformation, as well as the description on general target protein preparation mainly performed by the built-in tools associated with the docking software, whilst there were still some studies did not elaborate this domain.

For the method of target protein generation, further search from the RCSB Protein Data Bank revealed that the majority of the crystal structures used in the studies were obtained via X-ray diffraction method, where this method was more commonly in use compared to NMR spectroscopy. However, X-ray crystallography was ranked as “High Risk of Bias” in the checklist as it was opined that only NMR spectroscopy captures the three-dimensional structure details in a medium close to the actual biological environment [49]. Perhaps the ranking of this domain is worth revisiting since both NMR spectroscopy and X-ray crystallography offer different pros and cons, and both tools are highly complementary [102].

What is worth mentioning is almost all studies except two studies (data not found) performed visual control to inspect the docking pose to ensure there were no structural artifacts derived from the computer calculation. However, for docking validation by redocking, only a quarter of the studies elaborated on this crucial process. Since CADD methods are based on prediction by computational calculation and algorithm, experimental validation is still required to confirm the outcome of the study. Unfortunately, more than half of the studies did not pursue or yet report on the in-vitro validation.

In general, this assessment revealed that there is a gap in a proper or standard guide to enhance the reporting quality of studies involving molecular docking in particular. Nevertheless, Monks and colleagues have developed a 20-items checklist to improve the reporting of discrete-event simulation, system dynamics and agent-based simulation models within the field of Operational Research and Management Science, termed as “Strengthening The Reporting of Empirical Simulation Studies (STRESS)” [103]. However, the applicability of this checklist for this type of review is yet to be discovered.

## Limitation

One of the limitations of this review was that only articles written in English were included, resulting in missing important papers which were written in other languages. Besides, the included studies utilized different methods and approaches hence resulting in heterogenicity and difficulties in performing pooled analysis. On the other hand, there is no other systematic review that evaluated the anticancer drug design and discovery of anthraquinone derivatives based on CADD methods. Therefore, there is no comparison that could be done for assessing agreements or disagreements with other studies.

## Conclusion

The outcome of bias risk assessment implied the need for proper and more standardized guidelines in order to improve the reporting quality of in-silico studies. Nevertheless, the increasing number of publications retrieved throughout the years has proven the role of CADD methods as an indispensable tool in the era of modern drug design and discovery. These methods were particularly useful in the early stages of the drug design and discovery trajectory to screen, identify and optimize the potential hits in a systematic manner for shortlisting only the most promising compounds for further analysis.

Structure-based and ligand-based methods were either used alone or in combination to obtain consensus prediction. Combining different tools offered the advantage of enrichment enhancement, enabling the synergizing of the strengths and complementing the weaknesses of each method. The choices of software used were mainly project specific although user-friendly interface could have also served as one of the driving factors.

The findings in this review also strengthened the fact that CADD methods enabled deeper exploration of the anticancer potential of anthraquinone-based compounds up to the molecular level. The utilization of in-silico techniques for the study of anthraquinone derivatives has made it possible to obtain further insights into their structural, biological and pharmacological properties. Notably, anthraquinone derivatives demonstrated remarkable anticancer properties by targeting a wide spectrum of biological targets that tackled the abnormalities of cancer cells in an all-rounded fashion. The synergy between computational and experimental approaches contributes to a more comprehensive understanding of anthraquinones’ potential as anticancer therapeutics.

As cancer continues to pose a threat to the global healthcare system, the role of anthraquinones, coupled with CADD methods, offers a promising avenue for drug discovery. By harnessing the power of computational tools and leveraging the natural diversity of anthraquinone compounds, researchers can expedite the development of better drug to address the unmet medical needs in cancer treatment by improving the treatment outcome for cancer patients.

The insights gained from this review can serve as the scientific evidence-based guidance to improve the success rate of future cancer research. It is recommended for upcoming research to follow closely with the rapid advancement of CADD and make full use of integrated tools to facilitate the design and discovery of novel anticancer therapeutics expanded from the privileged anthraquinone scaffold.

## Supporting information

S1 Checklist PRISMA 2020 checklist. (DOCX)

S1 Fig Chemical structure of 9,10-anthraquinone scaffold. (TIF)

S1 Table Example of search strategy in PubMed. (DOCX)

S2 Table Data extraction form. (DOCX)
