# Supplementary figures and images for: Insights into the computer-aided drug design and discovery based on anthraquinone scaffold for cancer treatment: A systematic review
Source: PLoS One. 2024 May 22;19(5):e0301396. doi: 10.1371/journal.pone.0301396 (PMC11111074; doi:10.1371/journal.pone.0301396)

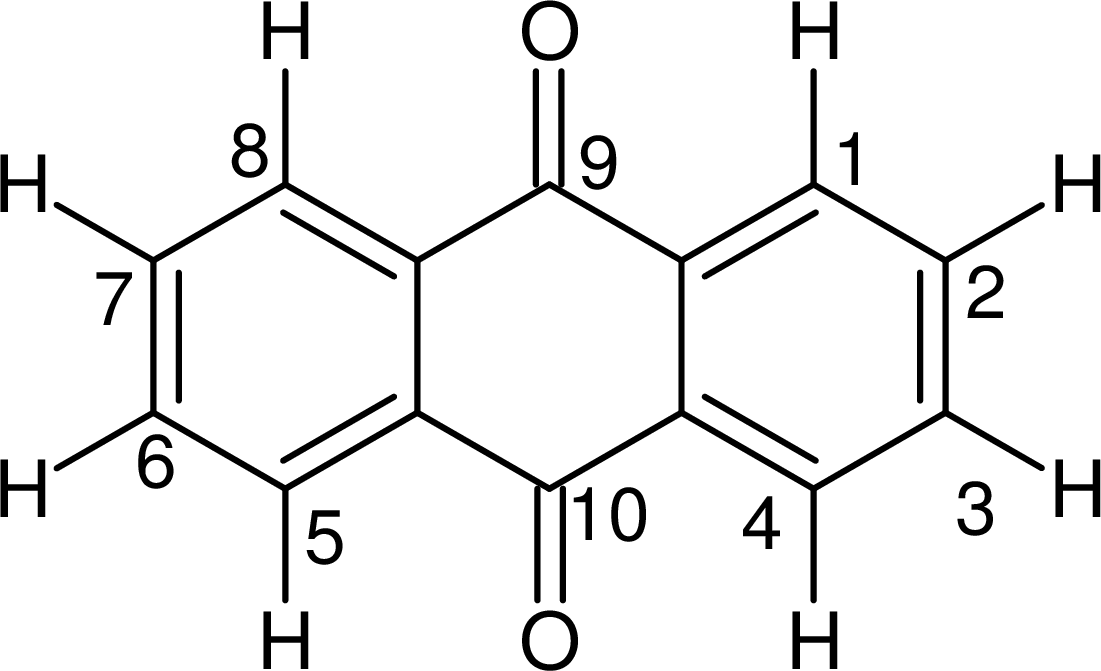

Supplement: S1 Fig — (TIF) [file pone.0301396.s002.tif]
