# Supplementary material for: Insights into the computer-aided drug design and discovery based on anthraquinone scaffold for cancer treatment: A systematic review
Source: PLoS One. 2024 May 22;19(5):e0301396. doi: 10.1371/journal.pone.0301396 (PMC11111074; doi:10.1371/journal.pone.0301396)
Supplement: S1 Table — (DOCX) [file pone.0301396.s003.docx]

**S1 Table. example of search strategy in PubMed**

| **Search** | **Query** |
| --- | --- |
| #1 | **"computer aided drug design"[Title/Abstract] OR "virtual screening"[Title/Abstract] OR "molecular docking"[Title/Abstract] OR "molecular dynamics"[Title/Abstract]** |
| #2 | “Anthraquinones"[MeSH Terms] |
| #3 | "anthraquinone*"[Title/Abstract] OR "anthracenedione*"[Title/Abstract] OR "anthranoid*"[Title/Abstract] OR "dioxoanthracene"[Title/Abstract] |
| #4 | "anthracene-9,10-dione"[Title/Abstract:~0] OR "anthracene-9,10-quinone"[Title/Abstract:~0] OR "9,10-anthrachinon"[Title/Abstract:~0] OR "9,10-dihydro-9,10-dioxoanthracene"[Title/Abstract:~0] |
| #5 | #2 OR #3 OR #4 |
| #6 | "Neoplasms"[MeSH Terms] |
| #7 | "Cancer"[Title/Abstract] OR "tumour"[Title/Abstract] OR "tumor"[Title/Abstract] OR "malignant"[Title/Abstract] OR "neoplasm"[Title/Abstract] |
| #8 | #6 OR #7 |
| #9 | #1 AND #5 AND #8 |
