# Supplementary material for: Insights into the computer-aided drug design and discovery based on anthraquinone scaffold for cancer treatment: A systematic review
Source: PLoS One. 2024 May 22;19(5):e0301396. doi: 10.1371/journal.pone.0301396 (PMC11111074; doi:10.1371/journal.pone.0301396)
Supplement: S2 Table — (DOCX) [file pone.0301396.s004.docx]

**S2 Table. data extraction form**

| **Domain** | **Key items** |
| --- | --- |
| Study characteristics | Journal title |
|  | Name of authors |
|  | Publication year |
| Study context | Target identification/ validation |
|  | Hit identification |
|  | Hit-to-lead |
|  | Lead Optimization |
| Compounds | Starting compounds with anthraquinone scaffold |
|  | Identified hits with anthraquinone scaffold |
| Cancer type | General |
|  | Specific cancer |
| Ligand-based method (specify the software/ tools used) | Pharmacophore modelling & mapping |
|  | Similarity search |
|  | Quantitative-structure activity relationship (QSAR) |
|  | Scaffold hopping |
| Structure-based method (specify the software/ tool used) | Molecular docking |
|  | Pharmacophore modelling & mapping |
| Source/ Database | Macromolecular target (specify the investigated target) |
|  | Ligand/ virtual library |
| Others CADD methods (Specify the software/ tool used) | ADMET filter |
|  | Molecular dynamic simulation |
|  | MM-GBSA |
|  | MM-PBSA |
| Experimental validation | Yes (specify IC_50_/EC_50_/Kd/Ki wherever applicable) |
|  | No |
